# The challenge of accurately documenting bee species richness in agroecosystems: bee diversity in eastern apple orchards

**DOI:** 10.1002/ece3.1582

**Published:** 2015-08-05

**Authors:** Laura Russo, Mia Park, Jason Gibbs, Bryan Danforth

**Affiliations:** 1 Entomology Department, Cornell UniversityIthaca, New York, 14853; 2 Departments of Humanities and Integrated Studies, University of North DakotaGrand Forks, North Dakota, 58202; 3 Department of Biology, University of North DakotaGrand Forks, North Dakota, 58202; 4 Department of Entomology, Center for Integrated Plant Systems, Michigan State University578 Wilson Rd., Room 202B, East Lansing, Michigan, 48824

**Keywords:** Agroecosystem, apple orchard, bee diversity, biodiversity, ecosystem function, pollination services, sampling

## Abstract

Bees are important pollinators of agricultural crops, and bee diversity has been shown to be closely associated with pollination, a valuable ecosystem service. Higher functional diversity and species richness of bees have been shown to lead to higher crop yield. Bees simultaneously represent a mega-diverse taxon that is extremely challenging to sample thoroughly and an important group to understand because of pollination services. We sampled bees visiting apple blossoms in 28 orchards over 6 years. We used species rarefaction analyses to test for the completeness of sampling and the relationship between species richness and sampling effort, orchard size, and percent agriculture in the surrounding landscape. We performed more than 190 h of sampling, collecting 11,219 specimens representing 104 species. Despite the sampling intensity, we captured <75% of expected species richness at more than half of the sites. For most of these, the variation in bee community composition between years was greater than among sites. Species richness was influenced by percent agriculture, orchard size, and sampling effort, but we found no factors explaining the difference between observed and expected species richness. Competition between honeybees and wild bees did not appear to be a factor, as we found no correlation between honeybee and wild bee abundance. Our study shows that the pollinator fauna of agroecosystems can be diverse and challenging to thoroughly sample. We demonstrate that there is high temporal variation in community composition and that sites vary widely in the sampling effort required to fully describe their diversity. In order to maximize pollination services provided by wild bee species, we must first accurately estimate species richness. For researchers interested in providing this estimate, we recommend multiyear studies and rarefaction analyses to quantify the gap between observed and expected species richness.

## Introduction

Biodiversity encompasses both species richness and the number of species roles in the community (functional diversity), and is a critical component of ecosystem function and the provision of ecosystem services (Balvanera et al. 2006; Cardinale et al. 2006). The relationship between biodiversity and ecosystem services may be driven by facilitation among different species, complementarity in ecosystem function between species in a diverse community, or the fact that the probability of communities including better service providers increases as we add more species (Loreau and Hector 2001; Cardinale et al. 2002). In particular, there has been a strong focus on the relationship between diversity and ecosystem services provided by pollinators in agroecosystems (Klein et al. 2003; Hoehn et al. 2008; Blüthgen and Klein 2011; Carvalheiro et al. 2011; Garibaldi et al. 2011; Albrect et al. 2012; Rogers et al. 2014). Recent studies have shown strong support for this relationship, emphasizing the positive correlation between crop yield and functional bee diversity in pollinator-dependent crops (Hoehn et al. 2008; Albrect et al. 2012; Rogers et al. 2014; Martins et al. 2015).

Unfortunately, biodiversity is very difficult to document accurately, especially for diverse arthropod taxa, because species can be difficult to detect. Additionally, ecological communities, including plant–pollinator communities, tend to comprise few abundant and many rare species (Kunin and Gaston 1993; Olesen and Jordano 2002; Russo et al. 2011). This could be especially important with regard to functional diversity, particularly if the rare species comprise a distinct functional component of the community (Petchey and Gaston 2006). It is critical to invest resources into sampling thoroughly over a long time period in order to accurately document the diversity of an ecosystem and subsequently the functional contribution of diversity. However, there is no consensus on the length of time that is sufficient to describe species richness in taxa that exhibit temporal variation in community composition. In species-rich systems, such as the tropics, biodiversity is only possible to quantify accurately after extensive effort (Longino et al. 2002). Bees can be particularly challenging taxa to document effectively because they are diverse, cryptic, and exhibit substantial year-to-year variation (Wilson et al. 2008; Grundel et al. 2011).

**Figure 1:**
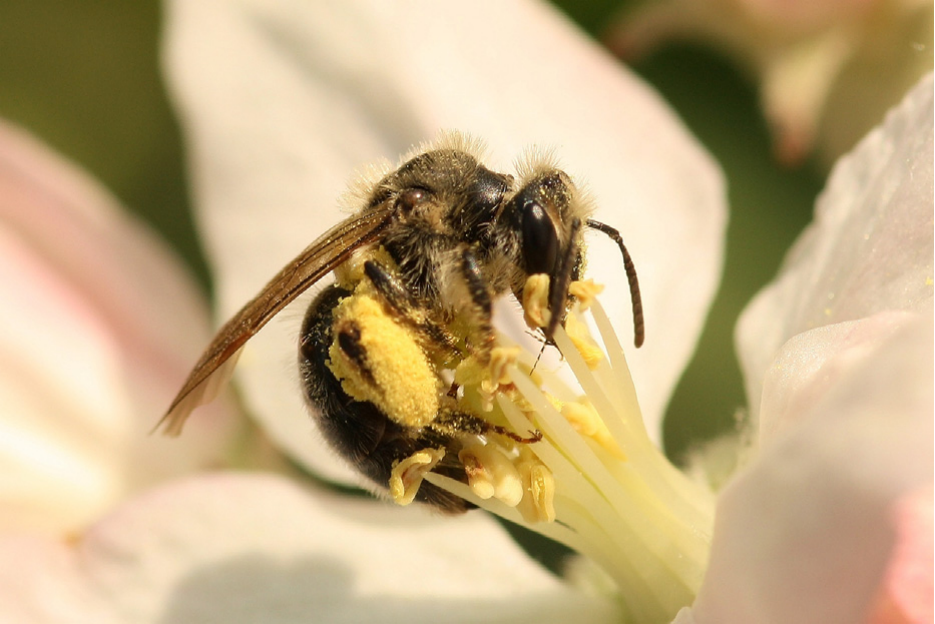
A wild mining bee, *Andrena mandibularis*, visiting an apple blossom in New York, USA.

In this study, we address the question of what constitutes sufficient sampling to elucidate the relationship between diversity and function in New York apple orchards. New York is the second largest producer of apples in the United States, and apple is an economically important crop for the region (USDA NASS 2013). In addition, apples are self-incompatible and require insect vectors for pollination; thus, a diversity of pollinator taxa, predominantly bees, is critical to crop yield (Free 1964; Garratt et al. 2014). Although peak apple bloom lasts just 2 weeks, apple represents a high quality resource that attracts a diversity of bee species (Fig.1; Gardner and Ascher 2006; Park et al. 2010; Watson et al. 2011; Sheffield et al. 2013; Mallinger and Gratton 2014). Maintaining the diversity of wild bees may be important to ensure adequate pollination services (Aizen and Harder 2009), but, to date, we do not have a rigorous, exhaustive survey of orchard bee diversity. For this reason, over a 6-year period, we conducted more than 760 unique sampling events in New York apple orchards during peak apple bloom.

To address the question of how much sampling is required to accurately document bee diversity within apple orchards during peak bloom, we used sample-based rarefaction plots. Using this method, we evaluate the degree of sampling required to fully document the diversity at individual orchards and across the study region and discuss reasons for variation in the amount of necessary sampling. Lessons learned from this study have implications for other studies of bee diversity and its relationship with pollination services in agroecosystems.

## Materials and Methods

**Figure 2:**
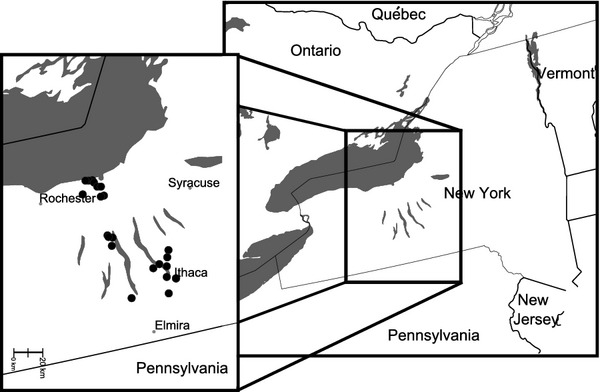
A map of the study sites (black circles) in the Finger Lakes region of western New York, USA.

We used aerial net collections to survey bee diversity in 28 apple orchards across the Finger Lakes region of western New York during peak bloom, which typically takes place over a period of 2 weeks in early to mid-May in this region (Fig.2). We collected bees on or near apple blossoms, as well as bees traveling between apple trees, on warm (>15°C), sunny days with low wind (sampling details are provided in Park et al. 2015). We used two sampling methods throughout the study. First, we conducted general collections, sampling only wild bees visiting apple trees with the goal of documenting the diversity of wild bees that may be pollinators of apple. The general collections lasted for a minimum of 15 min, but were sometimes longer. Second, we conducted standardized collections over a 15-min time period in which we collected all bees (both wild bees and honey bees) visiting apple trees along a 100-m transect. These standardized collections were meant to provide a comparable measure of abundance and diversity between different orchards. We first analyzed these two methods separately and then pooled them for the purpose of bee diversity assessment. We analyzed 22 of the sites individually and also the pooled samples from all 28 sites. We did not individually analyze the other six sites because we did not have a high enough sample size (i.e., there were fewer than 10 samples at these sites) to evaluate diversity independently in those orchards. Nonetheless, they contributed to our estimate of overall bee diversity in the region. The orchards varied in size from approximately 1–350 acres, and the number of samples per orchard ranged from 14 to 82 (Table1).

**Table 1:** Attributes of 22 orchards sampled across western New York as well as all sites pooled. The values in this table include standardized collections and general collections pooled and (standardized collections, general collections) separately. Orchards with a star (^*^) did not rent honeybee hives for the duration of the study period

Orchard	Size (acres)	Samples	Specimens	Obs species richness	Chao 1 mean (est richness)	Difference	Proportion
A^*^	1	16 (8, 8)	240 (137, 103)	36 (26, 29)	44.61 (27.79, 52.77)	8.61 (1.79, 23.77)	0.81 (0.94, 0.55)
B	1	14 (8, 6)	214 (95, 119)	33 (22, 29)	47.33 (42.04, 37.26)	14.33 (20.04, 8.26)	0.70 (0.52, 0.78)
C^*^	5	36 (20, 16)	597 (344, 253)	41 (35, 30)	52.98 (51.29, 41.95)	11.98 (16.29, 11.95)	0.77 (0.68, 0.72)
D	5	23 (16, 7)	515 (338, 177)	47 (40, 42)	54.13 (61.27, 54.43)	7.13 (21.27, 12.43)	0.87 (0.65, 0.77)
E^*^	10	36 (23, 13)	618 (464, 154)	42 (36, 24)	60.72 (50.05, 34.06)	18.72 (14.05, 10.06)	0.69 (0.72, 0.70)
F^*^	11	20 (13, 7)	128 (86, 42)	27 (18, 17)	58.75 (34.47, 46.53)	31.75 (16.47, 29.53)	0.46 (0.52, 0.37)
G	14	35 (23, 12)	437 (263, 174)	39 (30, 30)	45.04 (41.95, 57.96)	6.04 (11.95, 27.96)	0.87 (0.72, 0.52)
H	15	25 (17, 8)	398 (189, 209)	36 (32, 28)	42.38 (44.04, 36.96)	6.38 (12.04, 8.96)	0.85 (0.73, 0.76)
I^*^	15	46 (30, 16)	1279 (889, 190)	54 (45, 43)	79.58 (59.07, 74.92)	25.58 (14.07, 31.92)	0.68 (0.76, 0.57)
J	18	39 (32, 7)	559 (465, 94)	26 (24, 14)	41.97 (28.49, 16.64)	15.97 (4.49, 2.64)	0.62 (0.84, 0.84)
K	20	41 (23, 18)	514 (250, 264)	48 (36, 43)	104.14 (53.93, 74.88)	56.14 (17.93, 31.88)	0.46 (0.67, 0.57)
L	22	25 (14, 11)	321 (191, 130)	26 (22, 19)	41.95 (57.81, 19.74)	15.95 (35.81, 0.74)	0.62 (0.38, 0.96)
M	30	53 (38, 15)	848 (698, 150)	51 (47, 32)	83.36 (77.04, 43.92)	32.36 (30.04, 11.92)	0.61 (0.61, 0.73)
N	32	27 (18, 9)	120 (88, 32)	30 (23, 19)	46.2 (32.97, 27.07)	16.2 (9.97, 8.07)	0.65 (0.70, 0.70)
O	35	31 (23, 8)	477 (328, 149)	43 (35, 31)	51.62 (40.77, 37.7)	8.62 (5.77, 6.7)	0.83 (0.86, 0.82)
P^*^	50	23 (16, 7)	628 (416, 212)	42 (35, 29)	50.99 (49.05, 35.22)	8.99 (14.05, 6.22)	0.82 (0.71, 0.82)
Q	65	82 (65, 17)	1359 (1139, 220)	49 (46, 30)	67.74 (71.58, 38.06)	18.74 (25.58, 8.06)	0.72 (0.64, 0.79)
R	65	25 (15, 10)	177 (116, 61)	28 (21, 20)	70.01 (33.39, 90.82)	42.01 (12.39, 70.82)	0.40 (0.63, 0.22)
S	100	34 (27, 7)	417 (388, 29)	15 (10, 9)	23.15 (11.5, 15.03)	8.15 (1.5, 6.03)	0.65 (0.87, 0.60)
T	125	36 (26, 10)	370 (284, 86)	37 (28, 23)	49.22 (42.35, 33.01)	12.22 (14.35, 10.01)	0.75 (0.66, 0.70)
U	160	37 (24, 13)	267 (176, 91)	37 (25, 28)	44.17 (30.3, 41.85)	7.17 (5.3, 13.85)	0.84 (0.83, 0.67)
V	350	25 (18, 7)	343 (305, 38)	16 (11, 13)	21.98 (13.66, 17.38)	5.98 (2.66, 4.38)	0.73 (0.81, 0.75)
W	1264	760 (512, 248)	11219 (7888, 3331)	104 (91, 89)	118.14 (127.75, 95.53)	14.14 (36.75, 6.53)	0.89 (0.71, 0.93)

We identified all collected bee specimens to species using published revisions (Mitchell 1960, 1962; Ribble 1967, 1968; LaBerge 1969, 1971, 1973, 1977, 1980, 1987, 1989; LaBerge and Bouseman 1970; LaBerge and Ribble 1972; Bouseman and LaBerge 1979; McGinley 1986; Gibbs 2010, 2011; Rehan and Sheffield 2011; Gibbs et al. 2013) and comparison to expertly identified material deposited in the Cornell University Insect Collection (http://cuic.entomology.cornell.edu/). Voucher specimens were deposited in the Cornell University Insect Collection. We used Biota software for database management and exported the data into EstimateS for diversity analyses (version 9.1.0, Colwell 2013). Because our surveys were samples, and we did not collect any bees that were flying when we were not sampling (e.g., below 15°C or on rainy, cloudy, and windy days), we used the nonparametric Chao 1 estimator for species richness to develop rarefaction curves (Chao 1984). The number of expected species is modeled as a function of the number of samples and can tell us what proportion of the expected diversity was captured by our sampling at each locality (Table1). The Chao 1 log-linear confidence intervals are asymmetric and the lower bound cannot be lower than the observed number of species (Chao 1987). We compared the observed species richness to the mean Chao 1 estimator (between the lower and upper bounds). Using this estimate, we calculated a proportion of realized richness by dividing the observed number of species by the expected number of species given by the Chao 1 mean. For this reason, the true proportion of species richness observed could be over or underestimated, but the model gives us a reasonable expectation. Localities where the rarefaction curves have reached an asymptote have been sampled sufficiently, whereas localities with sloping rarefaction curves indicate that further sampling is required to characterize the diversity of the bee fauna. Rarefaction methods allow for the standardization and comparison of diversity datasets (Gotelli and Colwell 2001).

We calculated the percent agriculture within a 1 km radius of the orchard center with ArcGIS and a CropScape data layer (ESRI 2011, Han et al. 2014). The major landscape types we defined as agriculture include corn, soybeans, wheat, and tree fruit (see Table S1 in supporting information for a comprehensive list). To determine whether the size of the orchard, the percent agriculture within a 1 km radius of the orchard, and the number of sampling events correlated with the species richness of a given orchard, we used linear mixed effect models. We also used linear mixed effect models to test whether size and percent agriculture were predictors of the difference between the number of observed and expected species. Because many of the orchards rent honeybee hives during apple bloom (see Table1), we also tested for a correlation between honeybee abundance and wild bee abundance in the orchards. In addition, we tested the variation between the community composition of samples within the orchards, between years, and across sites using a Bray–Curtis dissimilarity index.

## Results

Over the 6-year sampling period, we conducted a total of 760 sampling events and collected a total of 11,219 bee specimens, representing 104 species (Table S2, supporting information). The species included representatives of five bee families: Andrenidae, Apidae, Colletidae, Halictidae, and Megachilidae. Although the highest diversity of species was recorded in the family Halictidae (41 species), the greatest abundance of bees was in the family Andrenidae (comprising 48% of the specimens) (Table2). The single most abundant species was the honeybee (*Apis mellifera*), at approximately 35% of the total abundance. Sixteen of the orchards brought in rental honeybee hives during apple bloom. However, there was no correlation between honeybee abundance and wild bee abundance (*P* > 0.05, *R*^2^ = 0.06).

**Table 2:** Proportion of bee diversity in each of five families collected in apple orchards over 6 years

Family	Number of specimens	Number of species
Andrenidae	5287	31
Apidae	4756	24
Colletidae	168	1
Halictidae	631	41
Megachilidae	128	7

**Figure 3:**
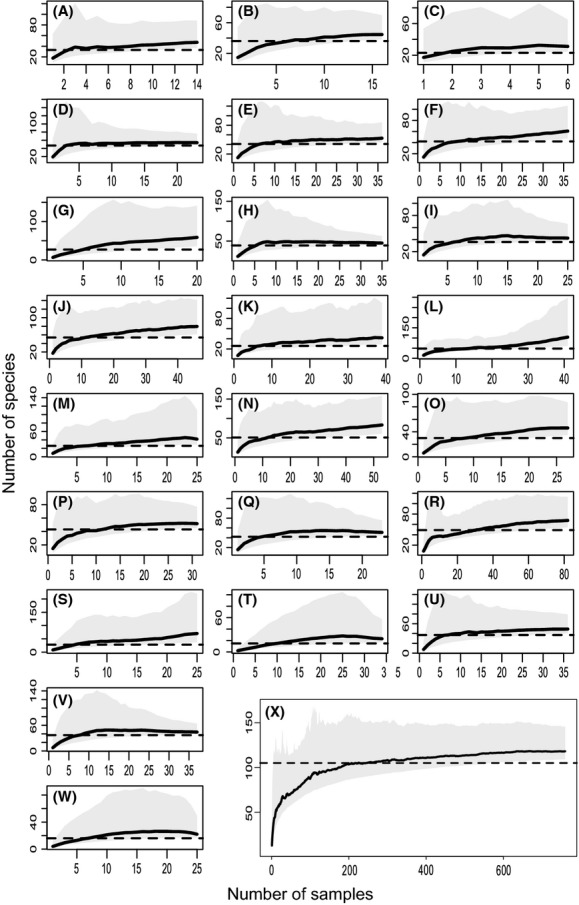
Rarefaction curves for the 22 orchards individually analyzed (A-V), as well as all sites pooled together (W). The dark gray area is the 95% confidence interval around the Chao 1 mean (solid line) for expected species richness as a function of sampling. The dashed line is the observed number of species. The letter in the upper right-hand corner refers to the name of the orchard; it refers to Table1 for a list of the orchard names. For 13 sites (marked by an X on the upper left hand corner of the graph), we captured <75% of the expected bee diversity (B, E, F, I, J, K, L, M, N, Q, R, S, and V). The rarefaction curve in eight of these 13 sites does not reach an asymptote (E, F, I, J, K, M, Q, and R).

Orchards varied in bee diversity, with a range from 15 to 54 observed bee species and an average of 36.5 ± 2.3 species (Fig.3). On average, we performed significantly more standardized than general collections in the orchards (*t*_21_ = 5.46, *P* < 0.01), and we collected significantly more specimens on average during standardized collections (*t*_21_ = 4.08, *P* < 0.01, Table1). Combined species richness from standardized and general collections was higher than species richness from standardized collections or general collections alone (*t*_21_ = 10.28, *P* < 0.01 for combined vs. general, *t*_21_ = 11.70, *P* < 0.01 for combined vs. standardized). On average, standardized collections resulted in higher observed species richness for orchards than general collections (*t*_21_ = 2.24, *P* < 0.05). Overall, when we pooled all sites together, the difference in species richness was small (91 species for standardized collections vs. 89 for general collections, Table1).

**Figure 4:**
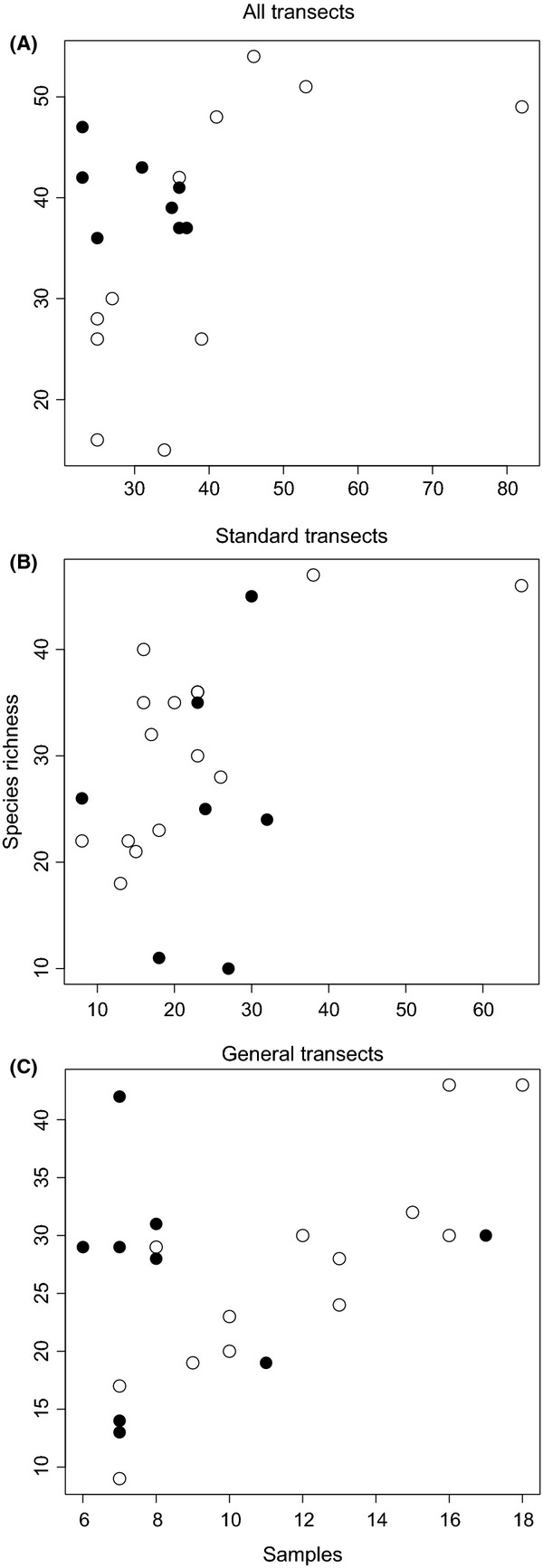
Scatter plots of the species richness versus the number of samples at each of the individually sampled sites for all collections combined (A), just the standardized collections (B), and just the general collections (C). There is a significant correlation between richness and samples for all collections combined (*P* < 0.05, *R*^2^ = 0.47), standardized collections (*P* < 0.05, *R*^2^ = 0.51), and general collections (*P* < 0.05, *R*^2^ = 0.52), but this correlation is not significant for orchards where we captured 75% or more of the expected species richness (dark circles) (*P* > 0.05).

When we controlled for the number of times we performed collections at each orchard, the size of the orchard was a significant predictor of observed species richness; larger orchards tended to have fewer species (*P* = 0.003). When we controlled for both number of collections and the size of the orchard, the percent agriculture in the surrounding landscape was also a significant predictor of observed species richness; more agriculture in the surrounding landscape had a negative effect on species richness (*P* = 0.01). Neither orchard size nor percent agriculture correlated with the difference between expected and observed species richness or proportion of realized species richness in the pooled methods, or either method individually (*P* > 0.05 for all tests). Nor was there a relationship between number of collections and proportion of expected richness collected in either type of collection. However, there was a correlation between the number of collections and the species richness of the orchards for all collections pooled (*P* < 0.05, *R*^2^ = 0.47), for standardized collections only (*P* < 0.05, *R*^2^ = 0.51), and for general collections only (*P* < 0.05, *R*^2^ = 0.52). This correlation was no longer significant when we excluded orchards where <75% of expected richness was captured (*P* > 0.05, Fig.4). In other words, in fully sampled orchards, there was no relationship between the number of samples and the number of species, as one would expect in a rarefaction curve that had reached an asymptote.

For all collections pooled, in 13 of 22 sites individually analyzed, we captured <75% of the expected richness. In 8 of these 13 sites, estimated species richness did not asymptote in their species accumulation curves, including our most heavily sampled site, which had been sampled 82 times (Fig.3Q). The lack of an asymptote of the curve means that each new sample is still leading to a higher estimate of species richness, whereas one might expect at a fully described site that new sampling events would not yield new species. At a regional level, with all 28 sites pooled, we captured almost 90% of the expected bee diversity, suggesting our regional sampling has been effective. In fact, the region as a whole was better sampled than any individual site, suggesting that the variation between sites was lower than the variation between samples. In other words, the bee fauna is relatively stable across the geographic region sampled. The highest ratio of observed to expected species richness (and hence the best sampling) for both methods together occurred in two orchards both at 87% (Fig.3D and G). On the other hand, we sampled one orchard (Fig.3R) 25 times across the 6 years and only recovered 40% of the expected species richness.

We captured 75% or more of the expected species richness in 7 sites using just standardized collections and 8 sites using just the general collections (Fig. S1). The proportion of expected species richness captured varied between 38 and 94% for standardized collections and 22 and 96% for general collections. Interestingly, the most poorly sampled orchard depended on the method used (although the most poorly sampled was orchard R for both general collections and all collections pooled); the same is true of the best sampled orchard for each method. Indeed, the worst sampled orchard of the standardized collections was the best sampled of the general collections (Table1L, Fig.3L).

In all but 6 of the 22 sites (B, D, J, K, L, and O), variation in community composition of bee species between years was higher than the variation between sites across the region, as measured by the Bray–Curtis dissimilarity index. At four of the six sites where the temporal variation was lower than the regional variation, the observed species richness was <75% of the expected species richness.

## Discussion

Our study entailed more than 190 h of sampling across 28 orchards distributed over an area of approximately 357,000 hectares during a 6-year period (2008–2013). For the region as a whole, we captured nearly 90% of the Chao 1 expected bee diversity, but within individual orchards, our sampling success varied. Despite the thoroughness of our sampling, the rarefaction curves for many of our sites did not achieve an asymptote (even in some heavily sampled sites; Fig.3), and in 13 of the 22 sites analyzed individually, we captured <75% of the expected species richness (Table1).

Our results demonstrate that, despite substantial sampling effort, it can be very challenging to thoroughly document pollinator diversity in agroecosystems. For some questions, a rough estimate of bee diversity may be sufficient, but for studies quantifying a relationship between biodiversity and ecosystem function, or for those attempting to elucidate community level structures, an accurate estimate of species richness could be essential (Gotelli and Colwell 2001; Balvanera et al. 2006). Poorly sampled sites may lead to a profound misunderstanding of community function and services. In particular, we cannot understand the relationship between the functional diversity of pollinators and the ecosystem service of pollination in agroecosystems without an accurate estimate of species richness (Balvanera et al. 2006). In addition, many recent studies have focused on applying network theory as a tool for understanding the function of pollinator communities (e.g., Blüthgen and Klein 2011; Russo et al. 2013; Winfree et al. 2014); however, such studies would be especially susceptible to insufficient sampling effort because network structure is heavily dependent on network size (number of species) (Dormann et al. 2009). It is therefore essential to use species richness estimators to determine how well a given site or ecosystem has been sampled and then to account for the potential gap between observed and expected species richness.

We still do not know why the amount of sampling required in individual sites varies; however, it is possible that the nature of the study system contributes. Bee populations can be highly variable and dependent on climatic conditions. For example, the abundance of univoltine bees is highly dependent on the previous year's population size and winter survival. The flowering season of apples is short, just 2–3 weeks a year. This means that the sampling window is short each year and there could be a high potential for phenological mismatch between individual bee species and the apple trees in any given year (but see Bartomeus et al. 2013). In addition, the timing of the sampling period (i.e., apple bloom) is in early May, which is early spring in central New York. Thus, the weather is poor on many days and further restricts the number of days for sampling as well as the activity of the bees, which may explain the pronounced year-to-year variation we observed. This means that for a highly variable fauna, we also have highly variable sampling.

We were able to show that there is a relationship between sampling and species richness for undersampled sites. For orchards in which we have realized <75% of the expected species richness, there is a correlation between species richness and the number of collections, but this relationship is not significant for orchards for which we have realized more than 75% of the expected species richness. This correlation is intuitive because, for undersampled sites, the rarefaction curve still has a positive slope. When it plateaus (generally above 75% of expected species richness), the correlation between sampling and species richness disappears. Neither orchard size nor percent agriculture in the surrounding landscape bears a relationship to the proportion of expected species richness in our apple orchards, so the mechanism driving the need for some orchards to be more heavily sampled is still unclear. The results of this study do agree with the heterogeneity required in sampling intensity in other systems, suggesting that sampling intensity should be considered in experimental design when the study taxa are diverse or dynamic (e.g., Shapiro et al. 2014). Temporal variation may drive some of the differences in the sampling required to fully describe the bee diversity in each orchard, although four of the six sites where the year-to-year variation was lower than the regional variation were undersampled. Variation in our ability to capture the expected species richness may also be due to more variable weather patterns at some sites, changes in the local landscape over the course of our study, or other differences in orchard management practices.

To the best of our knowledge, this is the most thorough existing study of bee diversity in apple orchards and perhaps one of the most rigorous of bee diversity in any agroecosystem. Despite the often broad geographic context of these studies, they tend to be restricted to a single year (but for notable exceptions with 3 year studies see Chacoff and Aizen 2006; Greenleaf and Kremen 2006; Tuell et al. 2009), while our results suggest that, for most sites, between-year variation is greater than between-site variation, at least for apple orchards. Our study also demonstrates the highest recorded bee diversity for apple orchards (another study shows higher richness [114 spp.] when multiple habitat types are included [Sheffield et al. 2013]). Reported values vary between 29 and 92 species. At the same time, because we collected samples of apple-visiting bee diversity, rather than entire bee populations, we will have necessarily missed some bees. The rarefaction analyses we employ help to quantify the difference between the diversity that we would expect and the diversity that we captured.

While many studies employ pan trapping (Sheffield et al. 2013; Mallinger and Gratton 2014), or aerial netting and pan trapping (Watson et al. 2011), our sampling was restricted to aerial netting. We targeted bee species that were actively foraging within apple orchards, which allowed us to focus on potential pollinators. Pan trapping may find a still greater bee species richness, as it could attract bees moving through the orchards but not actively foraging on apple (Shapiro et al. 2014). In other words, our study may represent a subset of the total background bee community. However, the species which do not visit apple blossoms are unlikely to be effective apple pollinators. In published studies of bee diversity in agroecosystems (reviewed in Kennedy et al. 2013), only the blueberries (Tuell et al. 2009) have a comparable bee diversity to the apple orchards in this study. Tuell et al. (2009) also used pan traps to capture specimens, which could include a background community of bees that are not visiting the blueberry flowers or actively pollinating the crop. Whether this disparity is due to true differences in bee diversity or differential sampling effort among studies is unknown.

## Conclusions

It is possible that accurate estimates of bee species richness could be necessary to quantify the relationship between diversity and function in an ecosystem. Rare species can be very important for ecosystem function (Lyons and Schwartz 2001) and can change the structure of the community (Vázquez et al. 2007). Thus, these estimates may be especially important when detailed community interactions are taken into consideration, such as in plant–pollinator networks. Greater sampling may change our perception of ecosystem function, including network structure. We have shown that the level of sampling required to get an accurate representation of bee diversity in an agroecosystem is often greater than would be expected and that year-to-year variation in these diverse taxa could result in deceptively low numbers of observed bee diversity. We recommend standardized, multiyear sampling protocols as well as sample-based rarefaction methods to assess sampling adequacy for future studies of pollinator diversity and pollination services in agroecosystems.

## Data Accessibility

Data from this manuscript will be made publicly available at the online database Dryad (http://datadryad.org/).

## Supporting Information

**Figure S1.** Rarefaction curves for the 22 orchards individually analyzed (A-V), excluding orchard C, where we only conducted standardized transects.

Table S1. Land cover types included in classification of agricultural areas. **Table S2.** List of bee species collected in the orchards, their abundance, and the number of orchards they were collected in.

## References

[b1] Aizen MA, Harder LD (2009). The global stock of domesticated honey bees is growing slower than agricultural demand for pollination. Curr. Biol.

[b2] Albrect M, Schmid B, Hautier Y, Müller CB (2012). Diverse pollinator communities enhance plant reproductive success. Proc. Biol. Sci.

[b3] Balvanera P, Pfisterer AB, Buchmann N, He J-S, Nakashizuka T, Raffaelli D (2006). Quantifying the evidence for biodiversity effects on ecosystem functioning and services. Ecol. Lett.

[b4] Bartomeus I, Park MG, Gibbs J, Danforth BN, Lakso AN, Winfree R (2013). Biodiversity ensures plant–pollinator phenological synchrony against climate change. Ecol. Lett.

[b5] Blüthgen N, Klein A-M (2011). Functional complementarity and specialisation: the role of biodiversity in plant–pollinator interactions. Basic Appl. Ecol.

[b6] Bouseman JK, LaBerge WE (1979). A revision of the bees of the genus *Andrena* of the Western Hemisphere. Part IX. Subgenus *Melandrena*. Trans. Am. Entomol. Soc.

[b7] Cardinale BJ, Palmer MA, Collins SL (2002). Species diversity enhances ecosystem functioning through interspecific facilitation. Nature.

[b8] Cardinale BJ, Srivastava DS, Emmett Duffy J, Wright JP, Downing AL, Sankaran M (2006). Effects of biodiversity on the functioning of trophic groups and ecosystems. Nature.

[b9] Carvalheiro LG, Veldtman R, Shenkute AG, Tesfay GB, Pirk CWW, Donaldson JS (2011). Natural and within-farmland biodiversity enhances crop productivity. Ecol. Lett.

[b10] Chacoff NP, Aizen MA (2006). Edge effects on flower-visiting insects in grapefruit plantations bordering premontane subtropical forest. J. Appl. Ecol.

[b11] Chao A (1984). Nonparametric estimation of the number of classes in a population. Scand. J. Stat.

[b12] Chao A (1987). Estimating the population size for capture-recapture data with unequal catchability. Biometrics.

[b13] Colwell RK (2013).

[b14] Dormann CF, Fründ J, Blüthgen N, Gruber B (2009). http://goedoc.uni-goettingen.de/goescholar/handle/1/5837.

[b15] ESRI (2011). ArcGIS desktop: release 10.

[b16] Free JB (1964). Comparison of the importance of insect and wind pollination of apple trees. Nature.

[b17] Gardner KE, Ascher JS (2006). Notes on the native bee pollinators in New York apple orchards. J. N. Y. Entomol. Soc.

[b18] Garibaldi LA, Steffan-Dewenter I, Kremen C, Morales JM, Bommarco R, Cunningham SA (2011). Stability of pollination services decreases with isolation from natural areas despite honey bee visits. Ecol. Lett.

[b19] Garratt MPD, Breeze TD, Jenner N, Polce C, Biesmeijer JC, Potts SG (2014). Avoiding a bad apple: insect pollination enhances fruit quality and economic value. Agric. Ecosyst. Environ.

[b20] Gibbs J (2010). Revision of the metallic species of *Lasioglossum**Dialictus*) in Canada (Hymenoptera, Halictidae, Halictini). Zootaxa.

[b21] Gibbs J (2011). Revision of the metallic *Lasioglossum**Dialictus*) of eastern North America (Hymenoptera: Halictidae: Halictini). Zootaxa.

[b22] Gibbs J, Packer L, Dumesh S, Danforth BN (2013). Revision and reclassification of *Lasioglossum**Evylaeus*), L. (*Hemihalictus*) and L. (*Sphecodogastra*) in eastern North America (Hymenoptera: Apoidea: Halictidae). Zootaxa.

[b23] Gotelli NJ, Colwell RK (2001). Quantifying biodiversity: procedures and pitfalls in the measurement and comparison of species richness. Ecol. Lett.

[b24] Greenleaf SS, Kremen C (2006). Wild bees enhance honey bees’ pollination of hybrid sunflower. Proc. Natl Acad. Sci. USA.

[b25] Grundel R, Frohnapple KJ, Jean RP, Pavlovic NB (2011). Effectiveness of bowl trapping and netting for inventory of a bee community. Environ. Entomol.

[b26] Han W, Yang Z, Di L, Yue P (2014). A geospatial Web service approach for creating on-demand Cropland Data Layer thematic maps. Trans. ASABE.

[b27] Hoehn P, Tscharntke T, Tylianakis JM, Steffan-Dewenter I (2008). Functional group diversity of bee pollinators increases crop yield. Proc. Biol. Sci.

[b28] Kennedy CM, Lonsdorf E, Neel MC, Williams NM, Ricketts TH, Winfree R (2013). A global quantitative synthesis of local and landscape effects on wild bee pollinators in agroecosystems. Ecol. Lett.

[b29] Klein A-M, Steffan–Dewenter I, Tscharntke T (2003). Fruit set of highland coffee increases with the diversity of pollinating bees. Proc. R. Soc. Lond. B Biol. Sci.

[b30] Kunin WE, Gaston KJ (1993). The biology of rarity: patterns, causes and consequences. Trends Ecol. Evol.

[b31] LaBerge WE (1969). A revision of the bees of the genus *Andrena* of the Western Hemisphere part II. *Plastandrena, Aporandrena, Charitandrena*. Trans. Am. Entomol. Soc.

[b32] LaBerge WE (1971). A revision of the bees of the genus *Andrena* of the Western Hemisphere. Part IV*. Scrapteropsis, Xiphandrena* and *Raphandrena*. Trans. Am. Entomol. Soc.

[b33] LaBerge WE (1973). A revision of the bees of the genus *Andrena* of the Western Hemisphere. Part VI. Subgenus *Trachandrena*. Trans. Am. Entomol. Soc.

[b34] LaBerge WE (1977). A revision of the bees of the genus *Andrena* of the Western Hemisphere. Part VIII. Subgenera *Thysandrena, Dasyandrena, Psammandrena, Rhacandrena, Euandrena, Oxyandrena*. Trans. Am. Entomol. Soc.

[b35] LaBerge WE (1980). A revision of the bees of the genus *Andrena* of the western hemisphere. Part X. Subgenus *Andrena*. Trans. Am. Entomol. Soc.

[b36] LaBerge WE (1987). A revision of the bees of the genus *Andrena* of the Western Hemisphere. Part XII. Subgenera *Leucandrena, Ptilandrena, Scoliandrena*, and *Melandrena*. Trans. Am. Entomol. Soc.

[b37] LaBerge WE (1989). A revision of the bees of the genus *Andrena* of the Western Hemisphere. Part XIII. Subgenera *Simandrena* and *Taeniandrena*. Trans. Am. Entomol. Soc.

[b38] LaBerge WE, Bouseman JK (1970). A revision of the bees of the genus *Andrena* of the Western Hemisphere. Part III. *Tylandrena*. Trans. Am. Entomol. Soc.

[b39] LaBerge WE, Ribble DW (1972). A revision of the bees of the genus *Andrena* of the Western Hemisphere. Part IV. *Gonandrena, Geissandrena, Parandrena, Pelicandrena*. Trans. Am. Entomol. Soc.

[b40] Longino JT, Coddington J, Colwell RK (2002). The ant fauna of a tropical rain forest: estimating species richness three different ways. Ecology.

[b41] Loreau M, Hector A (2001). Partitioning selection and complementarity in biodiversity experiments. Nature.

[b42] Lyons KG, Schwartz MW (2001). Rare species loss alters ecosystem function – invasion resistance. Ecol. Lett.

[b43] Mallinger RE, Gratton C (2014). Species richness of wild bees, but not the use of managed honeybees, increases fruit set of a pollinator-dependent crop. J. Appl. Ecol.

[b44] Martins KT, Gonzalez A, Lechowicz MJ (2015). Pollination services are mediated by bee functional diversity and landscape context. Agric. Ecosyst. Environ.

[b45] McGinley RJ (1986). Studies of Halictinae (Apoidea: Halictidae), I: revision of new world *Lasioglossum* curtis. Smithson. Contrib. Zool.

[b46] Mitchell TB (1960). Bees of the Eastern United States: volume I. N. C. Agric. Exp. Sta. Tech. Bull.

[b47] Mitchell TB (1962). Bees of the Eastern United States: volume II. N. C. Agric. Exp. Sta. Tech. Bull.

[b48] Olesen JM, Jordano P (2002). Geographic patterns in plant–pollinator mutualistic networks. Ecology.

[b49] Park MG, Orr MC, Danforth BN (2010). The role of native bees in apple pollination. N.Y. Fruit Quart.

[b50] Park MG, Blitzer EJ, Gibbs J, Losey JE, Danforth BN (2015). Negative effects of pesticides on wild bee communities can be buffered by landscape context. Proc. R. Soc. B.

[b51] Petchey OL, Gaston KJ (2006). Functional diversity: back to basics and looking forward. Ecol. Lett.

[b52] Rehan SM, Sheffield CS (2011). Morphological and molecular delineation of a new species in the *Ceratina dupla* species-group (Hymenoptera: Apidae: Xylocopinae) of eastern North America. Zootaxa.

[b53] Ribble DW (1967). The monotypic North American *Larandrena* of *Andrena* (Hymenoptera: Apoidea). Bull. Univ. Nebr. State Mus.

[b54] Ribble DW (1968). Revisions of two subgenera of *Andrena*
*Micrandrena* Ashmead and *Derandrena*, new subgenus (Hymenoptera: Apoidea. Bull. Univ. Nebr. State Mus.

[b55] Rogers SR, Tarpy DR, Burrack HJ (2014). Bee species diversity enhances productivity and stability in a perennial crop. PLoS ONE.

[b56] Russo L, Stehouwer R, Heberling JM, Shea K (2011). The composite insect trap: an innovative combination trap for biologically diverse sampling. PLoS ONE.

[b57] Russo L, DeBarros N, Yang S, Shea K, Mortensen D (2013). Supporting crop pollinators with floral resources: network-based phenological matching. Ecol. Evol.

[b58] Shapiro LH, Tepedino VJ, Minckley RL (2014). Bowling for bees: optimal sample number for ‘bee bowl’ sampling transects. J. Insect Conserv.

[b59] Sheffield CS, Kevan PG, Pindar A, Packer L (2013). Bee (Hymenoptera: Apoidea) diversity within apple orchards and old fields in the Annapolis Valley, Nova Scotia, Canada. Can. Entomol.

[b60] Tuell JK, Ascher JS, Isaacs R (2009). Wild Bees (Hymenoptera: Apoidea: Anthophila) of the Michigan highbush blueberry agroecosystem. Ann. Entomol. Soc. Am.

[b61] USDA, N (2013).

[b62] Vázquez DP, Melián CJ, Williams NM, Blüthgen N, Krasnov BR, Poulin R (2007). Species abundance and asymmetric interaction strength in ecological networks. Oikos.

[b63] Watson JC, Wolf AT, Ascher JS (2011). Forested landscapes promote richness and abundance of native bees (Hymenoptera: Apoidea: Anthophila) in Wisconsin apple orchards. Environ. Entomol.

[b64] Wilson JS, Griswold T, Messinger OJ (2008). Sampling bee communities (Hymenoptera: Apiformes) in a desert landscape: are pan traps sufficient?. J. Kansas Entomol. Soc.

[b65] Winfree R, Williams NM, Dushoff J, Kremen C (2014). Species abundance, not diet breadth, drives the persistence of the most linked pollinators as plant-pollinator networks disassemble. Am. Nat.

