# Supplementary figures and images for: The challenge of accurately documenting bee species richness in agroecosystems: bee diversity in eastern apple orchards
Source: Ecol Evol. 2015 Aug 5;5(17):3531–40. doi: 10.1002/ece3.1582 (PMC4567859; doi:10.1002/ece3.1582)

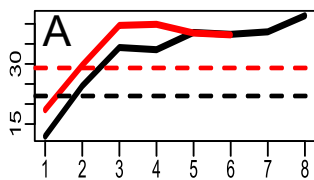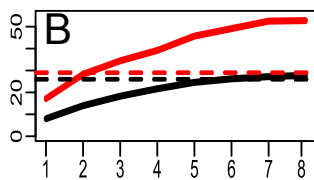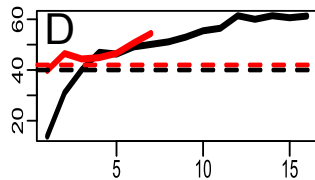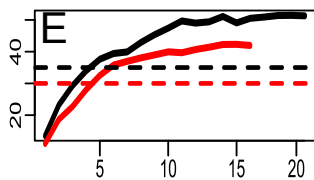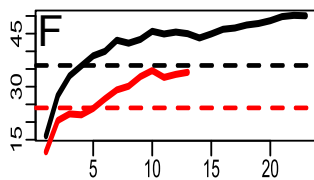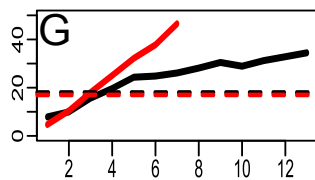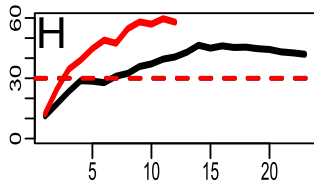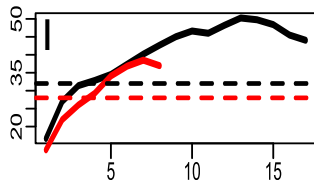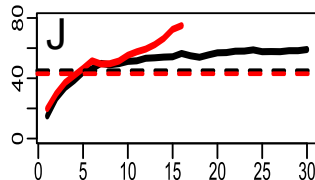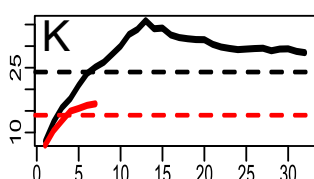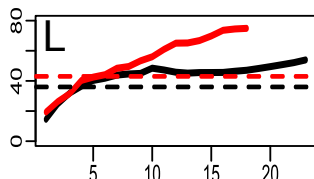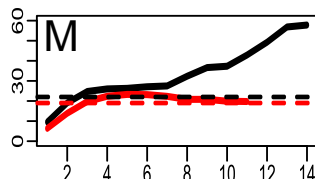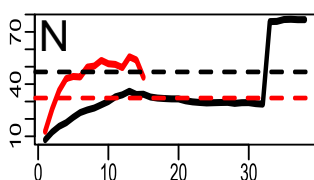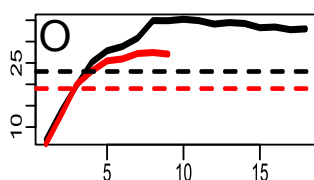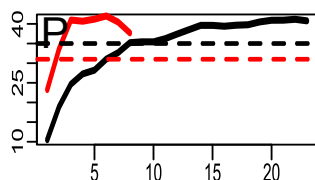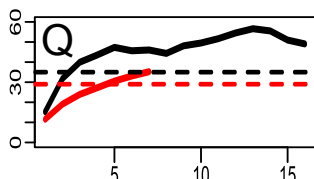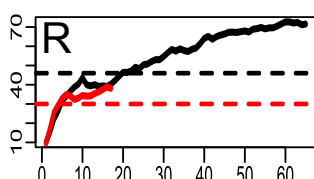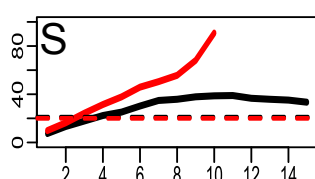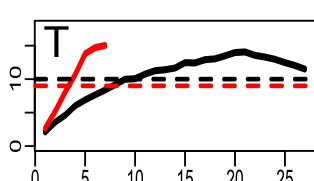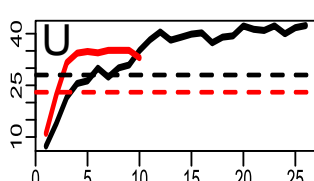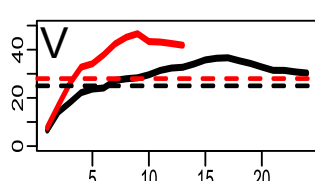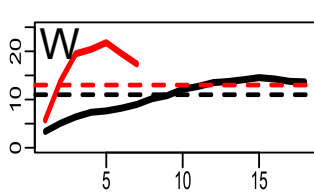

Supplement: Supplementary file 1 — Figure S1. Rarefaction curves for the 22 orchards individually analyzed (A-V), excluding orchard C, where we only conducted standardized transects. [file ece30005-3531-sd1.pdf]
