# Supplementary material for: The challenge of accurately documenting bee species richness in agroecosystems: bee diversity in eastern apple orchards
Source: Ecol Evol. 2015 Aug 5;5(17):3531–40. doi: 10.1002/ece3.1582 (PMC4567859; doi:10.1002/ece3.1582)
Supplement: Table S1. — Land cover types included in classification of agricultural areas. Table S2. List of bee species collected in the orchards, their abundance, and the number of orchards they were collected in. [file ece30005-3531-sd2.docx]

The challenge of accurately documenting bee species richness in agroecosystems: bee diversity in eastern apple orchards

Laura Russo, Mia Park, Jason Gibbs, Bryan Danforth

Supporting information. Figure 1. Rarefaction curves for the 22 orchards individually analyzed (A-V), excluding orchard C, where we only conducted standardized transects. The dashed line is the observed number of species and the solid lines are the expected number of species. The red lines are generalized transects and the black lines are the standardized transects. The letter in the upper right hand corner refers to the name of the orchard; refer to Table 1 for a list of the orchard names.

Table 1. Land cover types included in classification of agricultural areas. Mean percent composition of each class as well as their range (min-max) are provided within a 2km radius of study orchards (from Park et al 2015, with permission).

| **Class category** | **Cover type** | **Mean % composition (range)** |
| --- | --- | --- |
| **Agricultural** | Annual row crops | 15 (1.5 – 38.3) |
|  | Pasture/fallow fields | 11.5 (4.3 – 23.6) |
|  | Apple | 10.8 (0.2 – 38.0) |
|  | Perennial row crops | 6.3 (0.4 – 19.7) |
|  | Non-apple tree fruit | 0.5 (0.0 – 1.8) |
|  | Vineyard | 0.03 (0.0 – 0.1) |

Supporting information. Table 2. List of bee species collected in the orchards, their abundance, and the number of orchards they were collected in.

| Family | Species | Abundance | Number of Orchards |
| --- | --- | --- | --- |
| Andrenidae | *Andrena algida* Smith, 1853 *cf. nigrihirta* | 2 | 2 |
| Andrenidae | *Andrena barbilabris* (Kirby, 1802) | 18 | 10 |
| Andrenidae | *Andrena bisalicis* Viereck,1908 | 8 | 4 |
| Andrenidae | *Andrena carlini* Cockerell, 1901 | 219 | 26 |
| Andrenidae | *Andrena commoda* Smith, 1879 | 38 | 9 |
| Andrenidae | *Andrena crataegi* Robertson, 1893 | 1,367 | 23 |
| Andrenidae | *Andrena cressonii* Robertson, 1891 | 76 | 22 |
| Andrenidae | *Andrena dunning* Cockerell, 1898 | 45 | 14 |
| Andrenidae | *Andrena erythronii* Robertson, 1891 | 10 | 5 |
| Andrenidae | *Andrena forbesii* Robertson, 1891 | 161 | 22 |
| Andrenidae | *Andrena geranii* Robertson, 1891 | 3 | 1 |
| Andrenidae | *Andrena hippotes* Robertson, 1895 | 375 | 26 |
| Andrenidae | *Andrena imitatrix* Cresson, 1872 | 102 | 18 |
| Andrenidae | *Andrena mandibularis* Robertson, 1892 | 63 | 20 |
| Andrenidae | *Andrena milwaukeensis* Graenicher, 1903 | 46 | 15 |
| Andrenidae | *Andrena miserabilis* Cresson, 1872 | 354 | 23 |
| Andrenidae | *Andrena morrisonella* Viereck, 1917 | 31 | 13 |
| Andrenidae | *Andrena nasonii* Robertson, 1895 | 519 | 24 |
| Andrenidae | *Andrena nigrae* Robertson, 1905 | 1 | 1 |
| Andrenidae | *Andrena nivalis* Smith, 1853 | 5 | 4 |
| Andrenidae | *Andrena nuda* Robertson 1891 Robertson, 1891 | 4 | 4 |
| Andrenidae | *Andrena perplexa* Smith, 1853 | 131 | 24 |
| Andrenidae | *Andrena platyparia* Robertson, 1895 | 1 | 1 |
| Andrenidae | *Andrena pruni* Robertson, 1891 | 70 | 9 |
| Andrenidae | *Andrena regularis* Malloch, 1917 | 744 | 24 |
| Andrenidae | *Andrena rufosignata* Cockerell, 1902 | 1 | 1 |
| Andrenidae | *Andrena rugosa* Cockerell, 1906 | 181 | 25 |
| Andrenidae | *Andrena spiraeana* Robertson, 1895 | 2 | 1 |
| Andrenidae | *Andrena vicina* Smith, 1853 | 670 | 25 |
| Andrenidae | *Andrena wilkella* (Kirby, 1802) | 3 | 2 |
| Andrenidae | *Andrena w-scripta* Viereck, 1904 | 150 | 11 |
| Apidae | *Apis mellifera* Linnaeus, 1758 | 3,895 | 25 |
| Apidae | *Bombus bimaculatus* Cresson, 1863 | 57 | 16 |
| Apidae | *Bombus borealis* Kirby, 1837 | 1 | 1 |
| Apidae | *Bombus fervidus* (Fabricius, 1798) | 3 | 3 |
| Apidae | *Bombus griseocollis* (DeGeer, 1773) | 29 | 11 |
| Apidae | *Bombus impatiens* Cresson, 1863 | 426 | 26 |
| Apidae | *Bombus perplexus* Cresson, 1863 | 34 | 14 |
| Apidae | *Bombus sandersoni* Franklin, 1913 | 8 | 5 |
| Apidae | *Bombus ternaries* Say, 1837 | 7 | 5 |
| Apidae | *Bombus terricola* Kirby, 1837 | 2 | 2 |
| Apidae | *Bombus vagans* Smith, 1854 | 1 | 1 |
| Apidae | *Ceratina calcarata* Robertson, 1900 | 72 | 14 |
| Apidae | *Ceratina dupla* Say, 1837 | 4 | 3 |
| Apidae | *Ceratina mikmaqi* Rehan & Sheffield, 2011 | 1 | 1 |
| Apidae | *Nomada cressonii* Robertson, 1893 | 30 | 8 |
| Apidae | *Nomada denticulata* Robertson, 1902 | 1 | 1 |
| Apidae | *Nomada imbricata* Smith, 1854 | 3 | 2 |
| Apidae | *Nomada lehighensis* Cockerell, 1903 | 1 | 1 |
| Apidae | *Nomada luteoloides* Robertson, 1895 | 6 | 5 |
| Apidae | *Nomada maculata* Cresson, 1863 | 4 | 3 |
| Apidae | *Nomada media* Mitchell, 1962 | 1 | 1 |
| Apidae | *Nomada ovata* (Robertson, 1903) | 4 | 4 |
| Apidae | *Nomada pygmaea* Cresson, 1863 | 8 | 6 |
| Apidae | *Xylocopa virginica* (Linnaeus, 1771) | 270 | 26 |
| Colletidae | *Colletes inaequalis* Say, 1837 | 173 | 21 |
| Halictidae | *Agapostemon sericeus* (Förster, 1771) | 9 | 4 |
| Halictidae | *Agapostemon virescens* (Fabricius, 1775) | 1 | 1 |
| Halictidae | *Augochlora pura* (Say, 1837) | 74 | 17 |
| Halictidae | *Augochlorella aurata* (Smith, 1853) | 16 | 10 |
| Halictidae | *Augochloropsis metallica* (Fabricius, 1793) | 2 | 2 |
| Halictidae | *Halictus confusus* Smith, 1853 | 35 | 11 |
| Halictidae | *Halictus ligatus* Say, 1837 | 1 | 1 |
| Halictidae | *Halictus rubicundus* (Christ, 1791) | 43 | 12 |
| Halictidae | *Lasioglossum abanci* (Crawford, 1932) | 2 | 2 |
| Halictidae | *Lasioglossum atwoodi* Gibbs, 2010 | 2 | 2 |
| Halictidae | *Lasioglossum cinctipes* (Provancher, 1888) | 48 | 11 |
| Halictidae | *Lasioglossum coeruleum* (Robertson, 1893) | 17 | 2 |
| Halictidae | *Lasioglossum coriaceum* (Smith, 1853) | 2 | 2 |
| Halictidae | *Lasioglossum cressonii* (Robertson, 1890) | 10 | 7 |
| Halictidae | *Lasioglossum ephialtum* Gibbs, 2010 | 3 | 2 |
| Halictidae | *Lasioglossum foxii* (Robertson, 1895) | 36 | 14 |
| Halictidae | *Lasioglossum heterognathum* (Mitchell, 1960) | 1 | 1 |
| Halictidae | *Lasioglossum hitchensi* Gibbs, 2012 | 99 | 24 |
| Halictidae | *Lasioglossum imitatum* (Smith, 1853) | 4 | 3 |
| Halictidae | *Lasioglossum laevissimum* (Smith, 1853) | 22 | 10 |
| Halictidae | *Lasioglossum leucozonium* (Schrank, 1781) | 2 | 2 |
| Halictidae | *Lasioglossum lineatulum* (Crawford, 1906) | 22 | 9 |
| Halictidae | *Lasioglossum nigroviride* (Graenicher, 1911) | 1 | 1 |
| Halictidae | *Lasioglossum obscurum* (Robertson, 1892) | 16 | 5 |
| Halictidae | *Lasioglossum oceanicum* (Cockerell, 1916) | 3 | 3 |
| Halictidae | *Lasioglossum paradmirandum* (Knerer & Atwood, 1966) | 25 | 8 |
| Halictidae | *Lasioglossum pectorale* (Smith, 1853) | 1 | 1 |
| Halictidae | *Lasioglossum perpunctatum* (Ellis, 1913) | 5 | 3 |
| Halictidae | *Lasioglossum pilosum* (Smith, 1853) | 1 | 1 |
| Halictidae | *Lasioglossum planatum* (Lovell, 1905) | 3 | 3 |
| Halictidae | *Lasioglossum quebecense* (Crawford, 1907) | 40 | 10 |
| Halictidae | *Lasioglossum subviridatum* (Cockerell, 1938) | 1 | 1 |
| Halictidae | *Lasioglossum truncatum* (Robertson, 1901) | 14 | 5 |
| Halictidae | *Lasioglossum versans* (Lovell, 1905) | 7 | 4 |
| Halictidae | *Lasioglossum versatum* (Robertson, 1902) | 44 | 14 |
| Halictidae | *Lasioglossum weemsi* (Mitchell, 1960) | 7 | 6 |
| Halictidae | *Lasioglossum zephyrum* (Smith, 1853) | 3 | 3 |
| Halictidae | *Lasioglossum zonulum* (Smith, 1848) | 15 | 11 |
| Halictidae | *Sphecodes confertus* Say, 1837 | 1 | 1 |
| Halictidae | *Sphecodes cressonii* (Robertson, 1903) | 3 | 2 |
| Megachilidae | *Osmia atriventris* Cresson, 1864 | 2 | 1 |
| Megachilidae | *Osmia bucephala* Cresson, 1864 | 4 | 4 |
| Megachilidae | *Osmia conjuncta* Cresson, 1864 | 2 | 2 |
| Megachilidae | *Osmia cornifrons* (Radoszkowski, 1887) | 97 | 22 |
| Megachilidae | *Osmia lignaria* Say, 1837 | 5 | 4 |
| Megachilidae | *Osmia pumila* Cresson, 1864 | 17 | 8 |
| Megachilidae | *Osmia taurus* Smith, 1873 | 2 | 2 |
